# Physical examination findings and their relationship with performance-based function in adults with knee osteoarthritis

**DOI:** 10.1186/s12891-016-1151-3

**Published:** 2016-07-12

**Authors:** Maura D. Iversen, Lori Lyn Price, Johan von Heideken, William F. Harvey, Chenchen Wang

**Affiliations:** Department of Physical Therapy, Movement and Rehabilitation Sciences, Northeastern University, 360 Huntington Avenue 301 C RB, Boston, MA 02115 USA; Department of Medicine, Section of Clinical Sciences, Division of Rheumatology, Immunology & Allergy, Brigham & Women’s Hospital, Harvard Medical School, Boston, MA USA; Department of Women’s and Children’s Health, Karolinska Institute, Stockholm, Sweden; Institute of Clinical Research and Health Policy Studies, Tufts Medical Center, Boston, MA USA; Tufts Clinical and Translational Science Institute, Tufts University, Boston, MA USA; Center for Integrative Medicine and Division of Rheumatology, Tufts Medical Center, Tufts University School of Medicine, Boston, MA USA

**Keywords:** Knee osteoarthritis, Physical examination, Performance-based function, Self-reported function

## Abstract

**Background:**

Many physical examination (PE) maneuvers exist to assess knee function, none of which are specific to knee osteoarthritis (KOA). The Osteoarthritis Research Society International also recommends the use of six functional performance measures to assess function in adults with KOA. While earlier studies have examined the relationship between PE findings and self-reported function or PE findings and select performance tests in adults with knee pain and KOA, few have examined the all three types of measures. This cross-sectional study specifically examines the relationships between results of PE findings, functional performance tests and self-reported function in adults with symptomatic KOA.

**Methods:**

We used baseline PE data from a prospective randomized controlled trial in 87 participants aged ≥40 years with symptomatic and radiographic KOA. The PE performed by three experienced physical therapists included: muscle assessment, function and special tests. Participants also completed functional performance tests and the Western Ontario and McMaster Osteoarthritis Index (WOMAC). Multivariate linear regression identified contributions of PE findings towards functional performance and WOMAC scores, adjusting for age and gender.

**Results:**

Participants’ mean age was 60.4 years (SD = 10.5), mean disease duration was 8.4 years (SD = 10.1) and 27 participants had varus knee alignment. Mean WOMAC pain and function scores were 211 (SD = 113) and 709 (SD = 394), respectively. Weakness was present in major hip and knee muscles. Seventy-nine participants had a positive Ely’s, 65 a positive Waldron and 49 a positive Grind. Mean 6-min walk was 404 m (SD = 83) and mean Berg Balance was 53 (SD = 4). Regression analysis identified positive findings on 5 special tests (*P* < 0.05) as indicative of poorer 6 min walk. Positive Apley’s was associated (*P* < 0.05) with slower 20 m walk and a positive Ober with poorer balance scores (*P* < 0.05).

**Conclusions:**

Diminished hip muscle strength and flexibility, and patella dysfunction were prevalent in these adults with symptomatic KOA. Results of functional performance tests suggest balance and walking ability are impaired and are associated with PE findings of muscle length imbalance, hip muscle weakness and patella dysfunction. None of the PE measures were associated with self-reported function. Therefore, performance-based test results may be more useful in informing rehabilitation interventions.

## Background

Symptomatic knee osteoarthritis (KOA) is one of the ten most disabling diseases in developed countries affecting an estimated 19 % of women and 14 % of men in the United States over 45 years [1]. KOA results from progressive destruction of articular cartilage, ligaments, and joint capsule, synovial membrane inflammation and subchondral bone calcification [2]. Pain from knee and muscle impairments, mainly the quadriceps and hamstrings, are associated with KOA [3]. Other common symptoms of KOA include crepitus, reduced joint motion (both range of motion and arthrokinematic motion quality), impaired proprioception, joint line and periarticular tenderness on palpation and mild synovitis [2, 4–6]. These symptoms may produce impairments in body functions, activity limitations and participation restrictions [2]. In 2013, Osteoarthritis Research Society International (OARSI) provided a set of recommended performance-based measures to assess physical function in adults with KOA. These measures include tests of aerobic conditioning, walking speed, functional mobility and lower extremity strength [7].

Radiographic findings in the absence of a physical examination, are not very useful in identifying the source of pain in symptomatic KOA [8, 9]. Therefore, clinicians also rely on the patient history, physical examination (PE) procedures and special tests to assist in clinical decision-making. Special tests of muscle flexibility are used to assess muscle length and flexibility e.g., Ely’s for rectus femoris and Ober for the iliotibial band [10]. Ligamentous tests are conducted to examine knee joint integrity/stability, as a proxy for changes in knee biomechanics, secondary to muscular tightness or changes in lower limb alignment [10]. Despite the common use of these physical examination procedures in clinical practice, the psychometric properties of these procedures are weak [10, 11]. The poor association between PE findings and self-reported function and performance-based function may be related, in part, to central sensitization in arthritis, which can contribute to several of the “positive” findings. Performance based examinations are additional methods used to assess physical function and provide complementary information regarding KOA-associated disability [12]. Thus, research into optimal clinical examination sequence and composition is lacking and necessary given time constraints in clinical practice.

While studies have examined the association between PE findings and self-reported function in adults with knee pain [13] and in those with KOA [14–17], few studies have examined the association between PE findings and performance-based function in adults with symptomatic KOA [14]. This study provides a detailed clinical description of adults with symptomatic KOA and aims to determine the relative contribution of PE results primarily on performance-based function, adjusting for age and gender. Secondarily, we aimed to examine the contribution of PE findings on self-reported function (Western Ontario and McMaster Osteoarthritis Index (WOMAC) pain, WOMAC function). It was hypothesized that special tests of pain provocation, and muscle flexibility would more strongly correlate with functional performance than self-reported function and pain.

## Methods

This cross-sectional study is a secondary analysis of data from a prospective randomized clinical trial evaluating the effectiveness and cost-effectiveness of Tai Chi versus a physical-therapy regimen in adults with symptomatic and radiographically confirmed KOA [18, 19]. Institutional Human Subjects approval was obtained for this secondary study. We included the subset of 87 participants from the primary study who were randomized to physical therapy between January, 2010 and December, 2013 and for whom PE data existed.

### Recruitment and participants

The recruitment strategy has been described previously [18]. In brief, participants were recruited from the community using a multi-modal recruitment campaign. Study advertisements were placed in a variety of media venues including: myHospitalWebsite, Craigslist, Facebook, the medical center website, SAMPAN (a Chinese newspaper), local newspapers and via a booth at a senior expo. Participants were also identified from the rheumatology clinic patient database at a large urban tertiary medical center. Inclusion criteria for the primary trial consisted of adults aged 40 years or older with a diagnosis of symptomatic KOA based on the American College of Rheumatology criteria (pain on more than half the days of the past month during at least one of the following activities: walking, going up or down stairs, standing upright, or lying in bed at night) and who had radiographic evidence of tibiofemoral or patellofemoral OA (defined as the presence of a tibiofemoral compartment and/or patellofemoral compartment osteophyte in standing anterior/ posterior, lateral or skyline views) [20]. Weight-bearing radiographs were also scored using the Kellgren and Lawrence (K-L) grading system (range 0–4). A K-L grade of 0 indicates no radiographic features and higher grades indicate more severe global tibiofemoral radiographic structural damage [21]. Additionally, as the goal of the primary trial was to recruit participants with symptomatic KOA, all participants were required to have score of 40 or greater on the Western Ontario and McMaster’s Universities Osteoarthritis Index pain scale (WOMAC) [22, 23]. The study rheumatologist (WH) confirmed participant eligibility.

Participants were excluded if they: received physical therapy in the last year, had medical conditions affecting their ability to safely participate, received steroid injections or reconstructive surgery in the past three months, were non-English speakers, pregnant, had a score below 24 on the Mini-mental state exam [24], were non-ambulatory or had 100 % dependence on an assistive device, were enrolled in another clinical trial in last 30 days, or had plans to relocate. All participants were examined by one of three physical therapists (PTs). Human participants approval was obtained and all participants consented to participate.

### Study knee

For knee specific variables, we used data from the study knee, defined as the knee diagnosed with symptomatic KOA. For participants with bilateral KOA, we defined the study knee as the knee that was most severely affected according to WOMAC scores [22].

### Baseline intake

Participants completed a baseline intake with a study team member that included performance measures, a demographic survey (age, gender, race, co-morbidities, and marital/living status), Body mass index (BMI), and patient-reported outcome measures. Patient-reported knee-related outcome measures included the WOMAC pain and function scales, completed at the time of the clinical examination. The WOMAC (version VA3.1) is a reliable and valid instrument specifically designed to evaluate knee and hip OA [23]. The pain subscale score ranges from 0 to 500 and function scores from 0 to 1700, with higher scores indicating worse outcome. Performance tests included valid and reliable tests of gait speed, endurance, strength and balance as recommended by OARSI [7]. Gait speed was assessed with the timed 20-m (m) walk test. A research team member demonstrated the test procedure by walking the prescribed distance at a comfortable walking pace. The outcome was the total time taken to walk 20 m (m). Participants completed two trials and the average time to complete the trials was calculated [25]. The 6-min walk test, a reliable measure of functional exercise capacity, was used to evaluate endurance. Participants walked as fast and as far as possible within the six-minute period with verbal encouragement provided every minute. The distance, measured in meters, was recorded [26]. The Berg Balance Scale is a reliable test that assesses balance during the performance of 14 functional tasks such as standing from a seated position, standing unsupported for two minutes, turning 360°, and standing on one foot. Berg scores range from 0 to 56 with higher scores indicating better balance [27, 28]. The chair-stand test measured lower limb muscle strength and mobility. This test assesses the time taken to complete 10 full stands from a sitting position as fast as possible, and the fastest time of two trials was recorded to the nearest 0.01 s [29].

### Physical examination protocol

A supervising physical therapist (PT) research scientist (MDI) created a physical examination (PE) form based on contemporary orthopedic physical therapy practice. She conducted training sessions with the three study PTs to review the content of the PE and to develop a consensus on how to perform and document results of the special tests in a standardized manner. Specifically, the physical therapy team agreed upon a single method for each special test used for muscle flexibility, ligamentous and meniscal integrity and posture. The initial PE was performed the week following study intake by one of the three study PTs. All three PTs had ten or more years of clinical experience and one was also a certified Orthopedic Clinical Specialist. During each recruitment cycle, the supervising research scientist visited each therapist to ensure consistent documentation of examination procedures and for quality assurance.

The PE included past and current medical history and PE procedures. Selected comorbidities (Heart Disease, Hypertension and Diabetes) were collected via self-report. Patients were asked about the use of pain medication (classified as Nonsteroidal Anti-inflammatory Drugs (NSAIDs) or other type of analgesics), their chief complaint, and the duration, location and type of pain. Objective information included an integumentary assessment focused on edema, tenderness and sensation, joint range of motion, and muscle strength using manual muscle testing (MMT) [10]. The therapist observed gait and function and assessed the limb for the presence of a leg length discrepancy. An evaluation of knee contour and alignment was performed; specifically genu varus (>3 fingers apart at the knee with ankles together), valgus (>3.5 in. between malleoli when knees are together) or recurvatum (hyperextension of the knee joint greater than 0°), ankle alignment, pes planus or cavus. Next, special tests were used to assess muscle length of primary hip and knee musculature, such as the Ely’s Test and Ober Test [30, 31].

As KOA is influenced by knee biomechanics, tests of ligamentous integrity helped determine whether alterations in ligaments were directly affecting symptoms [32]. Ligamentous tests included were the Lachman’s, Posterior Sag and the Varus and Valgus Stress Tests at 0 and 30°. McMurray’s test was used to assess meniscal integrity [33]. The patellofemoral tests, Apley’s compression and distraction tests, the Grind and the Waldron tests, were included to assess patella dysfunction and tracking issues [34]. See Table 1 for details of PE procedures and reliability and validity information for these procedures. The final portion of the PE included a functional activity assessment with the PT noting whether an assistive device was used during ambulation.

**Table 1:** Standard physical exam procedures used for knee osteoarthritis

Test	Description of procedure and structure tested	Interrater reliability (Kappa)
Ely’s Test	Subject prone. Examiner stands next to the subject, at the side of the leg that will be tested. Examiner places one hand on lower back, the other holding the leg at the heel. The knee is passively flexed in a rapid fashion. The heel should touch the buttocks. Both sides are tested for symmetry. The test is positive when the heel cannot touch the buttocks, the hip of the tested side rises up from the table, or the patient feels pain or tingling in the back or legs. This procedure assesses the tightness of rectus femoris muscle.	0.46 [30]
Ober Test	Subject lies on the uninvolved side with hip and knee flexed at 90°. Examiner places the knee in 5° of flexion, fully abducts the leg being tested, and then allows gravity to adduct the extremity until the hip cannot adduct any further. The procedure assesses the tightness of the iliotibial band.	0.73 [47]
Lachman's	Examiner flexes the knee to 30° with the patient in supine and applies an anterior force to the tibia, noting any excess motion. The procedure assesses the integrity of the anterior cruciate ligament.	0.36 [48]
Posterior Sag	Examiner flexes the hip and knee to 90° with the patient and assess a possible posterior sag of the tibia. The test assesses the integrity of the posterior cruciate ligament.	Not found
Varus at 30°	The examiner brings the testing knee to 30° and applies a varus force while the subjects is in supine. The examiner notes any excess motion. The procedure assesses the lateral collateral ligament, the fibular collateral ligament and other posterior lateral corner knee structures.	Not found
Valgus at 0/30°	Examiner brings testing knee to 30° and applies a valgus force while the subject is in supine. The examiner notes any excess motion. Then repeats the test with the knee in 0°. This procedure assesses the medial collateral ligaments with or without the posterior capsule.	0.16 [49]
McMurray's	Subject supine. The examiner places one hand to the side of patella and other at distal tibia and extends the knee from maximum flexion to extension with internal rotation and varus stress. The knee is then returned to maximum flexion and the knee extended with external rotation and valgus stress. This procedure assesses meniscal integrity.	0.35 [50]
Apley's (distraction and compression)	Subject is prone with their knee flexed to 90°. The examiner medially and laterally rotates the tibia, combined first with distraction of the lower leg. The examiner then applies an axial load through the knee and rotates the joint via the lower leg. A positive test will result in pain or increased rotation relative to the other side when distraction is applied. Compression test – the test is positive if the rotation plus compression of patella is more painful or shows decreased rotation relative to the normal side. The distraction test assesses the medial and lateral collateral ligament. The compression test assesses meniscal integrity.	Not found
Waldron Sign	Examiner gently compresses the knee while the subject is squatting, noting any pain or crepitus. This procedure assesses patellofemoral joint and cartilage integrity. A positive test indicates the presence of chondromalacia, patella or anterior knee pain from patella contact pressure.	Not found
Grind Test	Subject supine with knee slightly flexed. The examiner provides distal force at superior border of patella as the patient contracts the quadriceps. Pain production indicates a positive test. The procedure assesses the integrity of the posterior patella and the trochlear groove of the femur.	Not found
Vastus Medialis Oblique Test	Patient supine, knee supported in 20° flexion. Patient actively extends knee. The examiner assesses contraction and movement of patella superiorly into grove. Graded as weak, no contraction or findings were within normal limits	Not found

### Statistical analysis

Descriptive statistics were used to characterize the sample. As most clinical special tests (e.g., Lachman, grind, etc.) are recorded as either positive or negative, these test results were treated as dichomotous variables. For tests that had more than 2-level responses, we collapsed them into dichotomous variables. Data from participants who completed 10 chair stands were included in analyses involving chair stands. T-tests were run to determine the association between the results of special tests and WOMAC scores and functional performance results. For the Ely test, a Wilcoxon Ranked Sum test was used to examine the association of this test with the Timed Chair Stand test.

In preparation for regression modeling, muscle weakness scores, by major muscle groups (ordinal scores ranged from 0 to 5) were collapsed into as a series of dichotomous variables (weakness defined as a score < 4) to indicate the presence of muscle weakness (y/n) in each major lower extremity muscle group. Scores for muscle weakness in the study leg were use in the regression. Cross tabulations of special tests for similar structures (e.g., ligament weakness) were conducted to examine the parsimony of the data to reduce variables in the modeling. Data were also examined to determine whether the occurrence of a positive test result was exceptionally low or exceptionally high (either < 10 % or >90 % positive test results) to determine appropriateness for statistical testing. Special tests with exceptionally low or exceptionally high positive test results were not included in data modeling. Multivariable linear regression was employed to model the primary outcomes: WOMAC function, WOMAC pain, 20 m walk, 6 Min walk, Timed Chair Stand test and Berg Balance with PE measures, adjusting for age and gender. Regression diagnostics were run on each model to determine the presence of influential observations. Results are presented for the whole cohort, however, when studentized residuals or Cook’s D indicated observations were influential, a comment on the impact of these observations on the regression is noted. The data analysis for this paper was generated using SAS software, version 9.4 for Windows (Copyright, SAS Institute Inc., Cary, NC, USA).

## Results

In the randomized controlled, 98 adults with KOA were recruited and randomized to physical therapy. Of these, 11 did not appear for the PE, leaving 87 participants who had complete PE data. Sixty participants (69 %) were female, the mean age was 60.4 years (SD = 10.5) and the mean duration of knee pain was 8.4 (SD = 10.1) years. Fifty-one (59 %) participants used NSAIDs and 28 (33 %) used other types of analgesics pain medication prior to the study to manage their knee symptoms. The majority of participants (57 %) were Caucasian and 47 (54 %) had a Body mass index (BMI) of ≥ 30 kg/m^2^, which is defined as being obese [25]. Eighty-one participants (91 %) had radiological structural findings of K-L grade 2 or more, indicating moderate to severe radiological structural knee damage. Twenty-four participants (28 %) presented with genu varus, 9 (10 %) with genu valgus and 4 (5 %) with recurvatum. Examination of the foot alignment revealed that 2 (2 %) had pes cavus and 49 (56 %) had pes planus. The average WOMAC pain score was 211 (SD = 113) and the average WOMAC function score was 709 (SD = 394). Of the 87 participants, 15 (17 %) ambulated with crutches/canes (Table 2).

**Table 2:** Demographic features, radiological characteristics, and self-reported outcomes in participants with knee osteoarthritis

Variable	Mean (SD)	Range
Age, years (*n* = 87)	60.4 (10.5)	41-85
Female, n (%) (*n* = 87)	60 (69 %)	
Race: n (%) (*n* = 87)		
Caucasian	50 (57 %)	
African American	27 (31 %)	
American Indian or Alaskan Native	3 (4 %)	
More than one race	4 (4 %)	
Other	3 (4 %)	
Mean BMI^a^ (kg/m^2^) (*n* = 87)	32 (6.9)	20-49
Duration of Knee Pain in index knee, years (SD) (*n* = 82)	8.4 (10.1)	<1-65
Mean Comorbidities (*n* = 87)		
Heart Disease	7 (8 %)	
Hypertension	42 (48 %)	
Diabetes	12 (14 %)	
NSAID^b^ prior to study, n (%) (*n* = 86)	51 (59 %)	
Other type of analgesics prior to study, n (%) (*n* = 86)	28 (33 %)	
Mean WOMAC^c^ pain at exam [0–500] (SD) (*n* = 84)	211 (113)	28-490
Mean WOMAC^c^ function at exam [0–1700] (SD) (*n* = 84)	709 (394)	47-1661

*SD* standard deviation

^a^BMI = Body mass index, ≥ 30 kg/m^2^ is defined as being obese

^b^NSAID = Nonsteroidal Anti-inflammatory Drugs

^c^WOMAC = Western Ontario and McMaster Universities. A self-administered, visual analogue scale specifically designed to evaluate knee and hip osteoarthritis. Higher scores indicating more severe disease symptoms

### Physical examination and performance data

Weak hip musculature was prevalent with the greatest impairments noted in hip flexors and extensors, followed by abductors/adductors. Thirty-seven participants (43 %) had weakness in the knee extensors and 21 (24 %) in the flexors. The mean Timed Chair Stand time was 31 s (SD = 11) and the average distance on the 6-min Walk Test was 404 m (SD = 83). The mean Berg Balance Score was 53 (SD = 4), indicating a low fall risk and the mean 20 m Walk was 18 s (SD = 3), indicating an average gait speed of 1.1 m/s (Table 3).

**Table 3:** Lower extremity muscle weakness of study knee and physical function scores in participants with knee osteoarthritis

Variable	
Hip flexor/extensor weakness, n (%) (*n* = 87/86)	46 (53) / 46 (53)
Hip abductor/adductor weakness, n (%) (*n* = 87/86)	52 (60) / 48 (56)
Hip internal/external rotator weakness, n (%) (*n* = 86/85)	17 (20) / 20 (24)
Knee flexor/extensor weakness, n (%) (*n* = 87/87)	37 (43) / 21 (24)
Ankle invertor/evertors weakness, n (%) (*n* = 87/87)	8 (9) / 8 (9)
Ankle dorsiflexor/plantarflexor weakness, n (%) (*n* = 87/87)	10 (11) / 8 (9)
Mean Timed Chair Stand^a^, seconds (SD) [range] (*n* = 78)	31 (11) [10–69]
Mean 6-min walk^b^, meters (SD) [range] (*n* = 84)	404 (83) [202–645]
Mean Berg Balance score^c^ (SD) [range] (*n* = 87)	53 (4) [35–56]
Mean time 20 m walk^d^, seconds (SD) [range] (*n* = 87)	18 (3) [10–32]

Muscle weakness defined as a Manual Muscle Test grade of ≥ 4 on scale from 0 to 5

Data is provided for all subjects who completed the test

^a^The test measures the time taken to complete 10 full stands from a sitting position as fast as possible

^b^The 6-min walk test measures the distance covered during the 6-min walk

^c^The Berg Balance Scale assessed balance during performance of 14 functional tasks, range from 0 to 56 and higher scores indicate better balance

^d^20 m walk test is the total time it takes to walk 20 m

### Results of special tests

Special tests for muscle flexibility indicated a prevalence of hip muscle inflexibility, especially in the rectis femoris (91 %). The results of the Grind and Waldron tests suggested many patients may have had patella-femoral joint involvement in addition to tibio-femoral KOA. Twenty-nine participants (34 %) had a positive McMurrary test of meniscal integrity. Nine participants (10 %) had a positive Lachman test which is one test to assess anterior cruciate ligament damage. No participants had a positive Posterior Sag and few had involvement of the medial collateral ligament or posterior crucial ligaments. These ligamentous test results indicate knee structures were relatively intact and the knees were stable (Table 4).

**Table 4:** Positive findings for special examinations performed on the study knee and performance test results

Clinical and Performance Tests	Number (%)
Vastus Medialis Oblique Weakness (*n* = 87)	75 (86)
Positive Ely’s (*n* = 87)	79 (91)
Positive Ober (*n* = 85)	29 (34)
Positive Lachmans Test (*n* = 86)	9 (10)
Positive Varus at 30° (*n* = 86)	2 (2)
Positive Valgus at 0/30° (*n* = 86)	16 (19)
Positive McMurray's (*n* = 86)	29 (34)
Positive Apley's (distraction) (*n* = 85)	5 (6)
Positive Apley's (compression) (*n* = 85)	15 (18)
Positive Waldron Sign (*n* = 87)	65 (75)
Positive Grind Test (*n* = 87)	49 (56)

### Univariate associations between special test results and WOMAC scores and functional performance

Using t-tests, special tests of ligamentous integrity, muscle flexibility and patella dysfunction were examined against the outcomes of performance tests and self-reported function and pain. Individuals with a positive Apley’s Compression test performed poorer (fewer cycles of rising and sitting) on the Timed Chair Test (mean difference of 8; *p* = 0.02). No other special tests were significantly associated with differences in performance-based outcomes. With respect to self-reported function, both WOMAC pain and function scores were significantly associated with a positive Apley’s Distraction test (mean difference of 103; *p* = 0.047 and mean difference of 498; *p* = 0.0006, respectively (Table 5).

**Table 5:** Difference in performance test findings and self-reported knee pain and function among those with positive and negative special examination results

	WOMAC pain^a^		WOMAC function^a^		20 m walk^b^		6 min walk^c^		Berg Balance^d^		Timed chair stand^e^	
Special Tests	Mean diff (95 % CI)	t-stat	Mean diff (95 % CI)	t-stat	Mean diff (95 % CI)	t-stat	Mean diff (95 % CI)	t-stat	Mean diff (95 % CI)	t-stat	Mean diff (95 % CI)	t-stat
Ely’s + = 79 - = 8	−52 (−135, 31)	−1.3	−247 (−535, 42)	−1.7	0 (−3, 2)	−0.3	41 (−24, 106)	1.2	−1 (−4, 2)	−0.7	+ Ely’s =29 –Ely’s =41.6 median	**N/A**
Lachman’s + = 9 - = 77	−26 −106, 53	−0.7	31 (−248, 311)	0.2	0 (−4,1)	−1.0	26 (−36, 88)	0.8	0 (−2, 3)	0.3	−5 (−12, 3)	−1.3
McMurray's + = 29 - = 57	32 (−21, 84)	1.2	158 (−24, 340)	1.7	−1 (−2, 1)	−0.6	−6 (−45, 33)	−0.3	−1 (−2, 1)	−0.6	−1 (−7, 4)	−0.5
Waldron + = 65 - = 22	−28 (−84, 29)	−1.0	−43 (−242, 155)	−0.4	−1 (−3, 1)	−1.3	23 (−19, 65)	1.1	0 (−2, 2)	0.3	−1 (−7, 5)	−0.3
Apley's (compression) + = 15 - = 70	29 (−36, 93)	0.9	113 (−111, 337)	1.0	−1 (−3, 1)	−1.2	36 (−9, 81)	1.6	1 (−2, 3)	0.7	−8 (−14, −1)	**−2.3**
Apley's (distraction) + =5 - = 80	103 (1, 205)	**2.0**	498 (150, 844)	**2.9**	3 (−1, 6)	1.6	−58 (−131, 14)	−1.6	−1 (−4, 3)	−0.3	8 (−4, 19)	1.3
Grind + = 49 - = 38	15 (−34, 65)	0.6	78 (−95, 250)	0.9	−1 (−2, 1)	−1.1	16 (−21, 52)	0.9	2 (0, 3)	1.7	−2 (−7, 3)	−0.8
Ober + = 29 - = 56	−49 (−98, 1)	−2.0	−163 (−340, 14)	−1.8	1 (−1, 2)	0.7	−22 (−61, 18)	−1.1	−1 (−3, 1)	−1.2	0 (−6, 5)	−0.1

+ number of positive test, − Number of negative test. *CI* confidence interval. Significant association (*P* < 0.05) in bold

^a^WOMAC = Western Ontario and McMaster Index

^b^20 m walk test is the total time it takes to walk 20 m

^c^The 6-min walk test measures the distance covered during the 6-min walk

^d^The Berg Balance Scale assessed balance during performance of 14 functional tasks

^e^The test measures the time taken to complete 10 full stands from a sitting position as fast as possible. Median values used to test Ely’s versus Timed Chair Stand

### Associations between physical examination findings and self-reported function and performance test scores adjusting for Age and gender

To determine the associations between PE findings, patient-reported outcomes and performance test results, after adjusting for age and gender, we conducted linear regression analysis (Table 6). There were no significant associations with self-reported pain (WOMAC) in the full regression. A positive Apley’s Distraction test (collateral ligament dysfunction) was significantly associated with lower self-reported function (WOMAC) (*p* < 0.01). Positive findings on five special tests (Ely’s, Waldron, Ober and Apley’s compression and distraction) were associated with 6-min walk test performance (*p* < 0.05 for each special test). A positive Ober test was also associated with poorer Berg Balance performance (*p* < 0.05). With respect to walking speed, a positive Apley’s distraction (collateral ligament dysfunction) was associated with slower 20 m walk times (*p* < 0.05). Individuals with a positive Ely’s test (tight rectus femoris) performed the Timed Chair test faster than those without a tight rectus femoris (*p* < 0.01). A positive Apley’s Compression test was also associated with better Timed Chair Test performance (*p* < 0.05).

**Table 6:** Association between outcomes of special tests, performance tests and self-reported knee pain and function

	WOMAC^a^ Pain number of observations = 80		WOMAC^a^ function number of observations = 80		20 m walk^b^ number of observations = 83		6 min walk^c^ number of observations = 80		Berg Balance^d^ number of observations = 83		Timed chair stand^e^ number of observations = 74	
	F = 1.74; p = 0.09 R^2^ = 0.09)		F = 2.27; p = 0.023 R^2^ = 0.14		F = 1.44; p = 0.18 R^2^ = 0.05		F = 1.93; p = 0.06 R^2^ = 0.11		F = 2.06; p = 0.04 R^2^ = 0.11		F = 1.93; p = 0.06 R^2^ = 0.11	
Explanatory variable	B	SE B	B	SE B	B	SE B	B	SE B	B	SE B	B	SE B
Positive Ely’s	−30.2	40.3	−175.3	138.9	−1.0	1.3	65.4*	31.6	−0.7	1.5	−12.7**	4.4
Positive Lachman’s	−8.9	37.9	109.3	130.5	−0.8	1.2	15.1	29.3	0.5	1.4	−3.7	3.9
Positive McMurray's	36.1	29.2	160.4	100.6	0.01	0.9	−24.9	21.4	−1.7	1.0	0.2	3.1
Positive Waldron sign	−56.3	28.9	−181.5	99.5	−1.4	0.9	44.7*	21.5	0.4	1.0	−2.1	3.0
Positive Apley’s (compression)	0.21	34.8	−66.3	120.0	−1.5	1.1	66.0*	25.8	0.5	1.3	−9.2*	3.7
Positive Apley’s (distraction)	86.9	52.0	489.3**	179.1	3.9*	1.7	−99.4*	38.3	−1.4	1.9	9.9	5.9
Positive Grind	6.1	26.1	75.0	90.0	0.01	0.8	0.7	19.3	1.7	0.9	0.1	2.7
Positive Ober	−21.6	27.8	−51.3	95.7	1.7	0.9	−44.7*	20.2	−2.2*	1.0	2.7	2.9
Age	−2.0	1.1	−2.0	3.9	0.02	0.04	−0.6	0.8	−0.1	0.04	0.1	0.1
Female	37.4	27.3	170.2	94.0	1.6	0.9	−20.3	20.1	−2.6**	1.0	0.9	2.9

Multivariate regression adjusted for age and sex. Significant association (*P* < 0.05) in bold

Notations for level of *p*-values as follows: * *p* < 0.05, ** *p* < 0.01

^**a**^WOMAC = Western Ontario and McMaster Index

^b^20 m walk test is the total time it takes to walk 20 m

^c^The 6-min walk test measures the distance covered during the 6-min walk

^d^The Berg Balance Scale assessed balance during performance of 14 functional tasks

^e^The test measures the time taken to complete 10 full stands from a sitting position as fast as possible

Influential points were identified for the following outcomes: WOMAC pain, Berg Balance score and the Timed Chair Stand. When the influential point was removed from the model with WOMAC pain as the outcome, a positive Apley’s Distraction test was found to be significantly associated with more self-reported knee pain, while a positive McMurray test was associated with an improvement in pain. With balance as the outcome, after removing one influential point, a positive McMurray test (meniscus pathology) was associated with poorer balance while a positive Grind test (assess integrity of posterior patella and the trochlear groove of the femur) was significantly associated with better balance. Two observations were found to be influential in the chair stand model. With these two observations removed, knee ligament dysfunction as noted by positive Apley’s Compression and Distraction test results, were associated with test performance, while the association with the positive Ely’s test became non-significant.

## Discussion

While earlier studies have examined the relationship between select PE findings and performance based function in adults with knee pain and others have examined self-reported function and PE findings or functional performance in adults with KOA [10–15], there is limited literature providing this level of detail on the clinical presentation of adults with symptomatic KOA and which address the relationship between self-reported function, performance-based function and special PE maneuvers. Recognizing there are no specific PE maneuvers to detect KOA, the procedures used in this study are typical PE maneuvers routinely used when evaluating knee function. Given the time constraints in clinical practice settings, this study focused on common PE special test maneuvers to determine whether these tests contributed to the variance in functional performance and self-report of function.

We demonstrated that lack of hip muscle flexibility is prevalent, especially in the rectus femoris and iliotibial band. These muscle groups may be less flexible due to biomechanical changes in the knee joint, knee pain, pain centralization and subsequent restriction of functional activities [35]. Patients in this sample also walked at slower speeds. According to Middleton et al., a walking speed of 1.1 m/s is considered to be lower than average, since normal walking speed in general is 1.2-1.4 m/s, not based on age or gender [36].

Hip muscle weakness, defined as a grade of ≥ 4 on MMT, of the hip flexors, extensors, abductors and adductors was present in more than 50 % of participants. These results are consistent with previous studies that demonstrate significant hip muscle weakness in individuals with KOA compared with asymptomatic controls [37–39]. Zeni et al. found lower extremity weakness in participants with end stage hip osteoarthritis is related to worse scores on functional performance tests whereas, hip pain is related to worse scores on questionnaires that capture self-perceptions of function [40]. There is a possibility that this is also true for participants with KOA.

Quadriceps muscle weakness is a well-documented clinical sign of KOA and has been attributed to activity limitations related to knee pain [3]. In this sample, 37 participants (43 %) had quadriceps weakness on manual examination and 21 (24 %) had hamstring weakness. The combination of special tests for patella femoral involvement were positive in more than half of the participants, suggesting knee arthritis was also affecting this area of the knee joint. Degenerative meniscus disease is a known component of KOA and we found one third of the participants had a positive McMurray’s test. Few participants had positive tests for ligamentous instability, although that is not unusual in a sample with older adults. Additionally, special tests for ligament instability have been reported to have poor inter-rater reliability (prevalence and bias-adjusted kappas of 0.02 to 0.3) and limited validity in this patient population. Thus, these tests may be less useful in clinical practice when examining patients with KOA [11].

With respect to self-reported outcomes, we found no significant associations between PE test results and self-reported pain and found only one significant association with self-reported function, a positive Apley’s test. Whereas, Wood et al. found moderate correlations between WOMAC function scores and specific PE tests, including: tenderness on palpation of the infrapatellar area, maximal isometric quadriceps femoris muscle strength, reproduction of symptoms on patellofemoral compression, and knee flexion range-of-motion. In this study, PE maneuvers explained between 7 and 13 % of the variance in WOMAC function scores, after controlling for age, gender, and body mass index [13]. The differences in our study results may be attributed in part to sample size, as Wood’s sample included over 800 individuals and this study included 87 adults. Additionally, Wood’s sample included adults with general knee pain while our sample consisted of adults with symptomatic KOA.

Evidence partially supported our hypothesis that special tests of muscle strength and flexibility would have greater association with performance test results than patient self-reported function and pain. We could determine the relative contribution of special test results on functional performance, specifically balance, functional exercise capacity and gait speed. For example, rectis femoris tightness was significantly associated with functional aerobic capacity (6 min Walk Test) and poorer balance (Berg test). A positive Waldron or squat test was not associated with patient-reported knee function although participants in this study were relatively high functioning, as indicated by their WOMAC function score. The lack of association between certain clinical maneuvers (special tests) and functional test performance may be explained by the fact that anatomical structures such as the meniscus and ligaments may be provoked with clinical maneuvers (forced rotation or anterior-posterior displacement) but are not stressed in the same manner with ambulation. Also, as special tests are designed to assess the integrity of specific anatomical structures, it is not surprising that a positive test for a single anatomical structure would not be associated with global self-reported knee function and pain. The lack of association between a positive Lachman’s test with functional performance is not surprising as participants with KOA often have stiff, stable knees and special tests for cruciate ligament integrity in adults with KOA have limited reliability and validity (Table 1) [11, 41].

In regression models, after adjusting for age and gender, there was some evidence that special tests of knee and hip structures, are associated with functional performance but less so with self-reported knee function and pain. These results are also in accordance with Tevald and colleagues who reported assessment of lower extremity strength is more closely associated with performance based test results than self-reported knee function and pain [37].

Our data suggest lower extremity muscle weakness and tightness in combination with knee pain were impacting walking endurance (6 Min Walk). Several studies have concluded that pain, muscle strength, obesity and age are more important determinants of functional impairment than radiological severity of KOA [42–44].

Our findings may have clinical implications. These preliminary data support the use of select PE procedures, muscle flexibility and strength of the hip and knee, when evaluating patients with KOA. The data suggest special tests of ligament integrity provide clinical information of little value. However, it is possible that the detection of any associations we found is limited by the range of PE procedures selected, and their corresponding validity and reliability in patients with KOA. We can only speculate, but we agree with Hinman et al., that there might be a benefit to include hip muscle strengthening into rehabilitation programs for patients with knee OA [39].

This study has a number of strengths. The performance-based outcome measures used (WOMAC, Berg etc.) are all valid and reliable measures with normative values available for patients with either KOA or of this age group. The PTs received formal instruction in the consistent application of the special tests and in the documentation of their findings to reduce reporting error and improve consistency of testing. Finally, patients were mixed with respect to race and socio-demographics to produce a more representative picture of adults with KOA.

Several limitations exist. The cross-sectional design does not allow for statements about cause and effect. The small sample might result in a type II error. There is also a potential for selection bias (WOMAC pain threshold for study entry), however, there was a relatively large variability in clinical presentations as well as the radiological severity of KOA. These participants could potentially have co-existing hip osteoarthritis, which could explain hip muscle weakness but this cannot be confirmed. There is also a potential for variability in the application of the PE maneuvers despite PT training and the use of a standard protocol. Additionally, manual muscle testing may not be sufficiently sensitive to measure strength compared to other more objective methods [45]. Finally, as we enrolled participants with bilateral KOA, individual’s perception of pain and functional ability may impact self-reported outcomes [46]. Hips were assessed by PTs (neurologic and joint range of motion). However, we did not collect additional PE data on hip (e.g., scour test) and lumbar in the comparative effectiveness trial.

## Conclusions

In conclusion, this study provides a rich clinical description of patient with symptomatic KOA. The results from this study are exploratory but suggest that hip muscle strength and flexibility need to be formally addressed in KOA management. The study also suggests that positive findings on clinical examination maneuvers are more strongly associated with functional performance tests than patient self-report of knee pain and function. Although, special tests of ligaments may provide little information of clinical value, as supported by studies of their reliability and validity [11, 41]. We propose the use of functional performance tests during the physical examination, when positive findings on special tests are present and as the use of these performance-based may better inform physical therapy interventions.

## Abbreviations

BMI, body mass index; KOA, knee osteoarthritis; MMT, manual muscle testing; NSAIDs, nonsteroidal anti-inflammatory drugs; OARSI, Osteoarthritis Research Society International; PE, physical examination; PTs, physical therapists; SD, standard deviation; WOMAC, Western Ontario and McMaster Osteoarthritis Index

## References

[1] Jordan JM, Helmick CG, Renner JB, Luta G, Dragomir AD, Woodard J, Fang F, Schwartz TA, Abbate LM, Callahan LF (2007). Prevalence of knee symptoms and radiographic and symptomatic knee osteoarthritis in African Americans and Caucasians: the Johnston County Osteoarthritis Project. J Rheumatol.

[2] Bijlsma JW, Berenbaum F, Lafeber FP (2011). Osteoarthritis: an update with relevance for clinical practice. Lancet.

[3] Bennell KL, Hunt MA, Wrigley TV, Lim BW, Hinman RS (2008). Role of muscle in the genesis and management of knee osteoarthritis. Rheum Dis Clin North Am.

[4] Walker J (2011). Effective management strategies for osteoarthritis. Br J Nurs.

[5] Knoop J, Steultjens MP, van der Leeden M, van der Esch M, Thorstensson CA, Roorda LD, Lems WF, Dekker J (2011). Proprioception in knee osteoarthritis: a narrative review. Osteoarthritis Cartilage.

[6] Baczkowicz D, Majorczyk E (2014). Joint motion quality in vibroacoustic signal analysis for patients with patellofemoral joint disorders. BMC Musculoskelet Disord.

[7] Dobson F, Hinman RS, Roos EM, Abbott JH, Stratford P, Davis AM, Buchbinder R, Snyder-Mackler L, Henrotin Y, Thumboo J (2013). OARSI recommended performance-based tests to assess physical function in people diagnosed with hip or knee osteoarthritis. Osteoarthritis Cartilage.

[8] Kornaat PR, Bloem JL, Ceulemans RY, Riyazi N, Rosendaal FR, Nelissen RG, Carter WO, Hellio Le Graverand MP, Kloppenburg M (2006). Osteoarthritis of the knee: association between clinical features and MR imaging findings. Radiology.

[9] Bedson J, Croft PR (2008). The discordance between clinical and radiographic knee osteoarthritis: a systematic search and summary of the literature. BMC Musculoskelet Disord.

[10] Cook C, Hegedus EJ (2008). Orthopedic physical examination tests : an evidence-based approach.

[11] Cibere J, Bellamy N, Thorne A, Esdaile JM, McGorm KJ, Chalmers A, Huang S, Peloso P, Shojania K, Singer J (2004). Reliability of the knee examination in osteoarthritis: effect of standardization. Arthritis Rheum.

[12] Stratford PW, Kennedy DM (2006). Performance measures were necessary to obtain a complete picture of osteoarthritic patients. J Clin Epidemiol.

[13] Wood L, Peat G, Thomas E, Hay EM, Sim J (2008). Associations between physical examination and self-reported physical function in older community-dwelling adults with knee pain. Phys Ther.

[14] Schmitt LC, Fitzgerald GK, Reisman AS, Rudolph KS (2008). Instability, laxity, and physical function in patients with medial knee osteoarthritis. Phys Ther.

[15] Kim I, Kim HA, Seo YI, Song YW, Hunter DJ, Jeong JY, Kim DH (2010). Tibiofemoral osteoarthritis affects quality of life and function in elderly Koreans, with women more adversely affected than men. BMC Musculoskelet Disord.

[16] Osaki M, Tomita M, Abe Y, Ye Z, Honda S, Yoshida S, Shindo H, Aoyagi K (2012). Physical performance and knee osteoarthritis among community-dwelling women in Japan: the Hizen-Oshima Study, cross-sectional study. Rheumatol Int.

[17] Adegoke BO, Babatunde FO, Oyeyemi AL (2012). Pain, balance, self-reported function and physical function in individuals with knee osteoarthritis. Physiother Theory Pract.

[18] Wang C, Iversen MD, McAlindon T, Harvey WF, Wong JB, Fielding RA, Driban JB, Price LL, Rones R, Gamache T (2014). Assessing the comparative effectiveness of Tai Chi versus physical therapy for knee osteoarthritis: design and rationale for a randomized trial. BMC Complement Altern Med.

[19] Wang C, Schmid CH, Iversen MD, Harvey WF, Fielding RA, Driban JB, Price LL, Wong JB, Reid KF, Rones R, McAlindon T. Comparative Effectiveness of Tai Chi Versus Physical Therapy for Knee Osteoarthritis: A Randomized Trial. Ann Intern Med. 2016. doi:10.7326/M15-2143. [Epub ahead of print]. 10.7326/M15-2143 PMC4960454 27183035

[20] Altman R, Asch E, Bloch D, Bole G, Borenstein D, Brandt K, Christy W, Cooke TD, Greenwald R, Hochberg M (1986). Development of criteria for the classification and reporting of osteoarthritis. Classification of osteoarthritis of the knee. Diagnostic and Therapeutic Criteria Committee of the American Rheumatism Association. Arthritis Rheum.

[21] Kellgren JH, Lawrence JS (1957). Radiological assessment of osteo-arthrosis. Ann Rheum Dis.

[22] Roos EM, Klassbo M, Lohmander LS (1999). WOMAC osteoarthritis index. Reliability, validity, and responsiveness in patients with arthroscopically assessed osteoarthritis. Western Ontario and MacMaster Universities. Scand J Rheumatol.

[23] Bellamy N, Kirwan J, Boers M, Brooks P, Strand V, Tugwell P, Altman R, Brandt K, Dougados M, Lequesne M (1997). Recommendations for a core set of outcome measures for future phase III clinical trials in knee, hip, and hand osteoarthritis. Consensus development at OMERACT III. J Rheumatol.

[24] Folstein MF, Folstein SE, McHugh PR (1975). "Mini-mental state". A practical method for grading the cognitive state of patients for the clinician. J Psychiatr Res.

[25] Motyl JM, Driban JB, McAdams E, Price LL, McAlindon TE (2013). Test-retest reliability and sensitivity of the 20-meter walk test among patients with knee osteoarthritis. BMC Musculoskelet Disord.

[26] Guyatt GH, Sullivan MJ, Thompson PJ, Fallen EL, Pugsley SO, Taylor DW, Berman LB (1985). The 6-minute walk: a new measure of exercise capacity in patients with chronic heart failure. Can Med Assoc J.

[27] Jogi P, Spaulding SJ, Zecevic AA, Overend TJ, Kramer JF (2011). Comparison of the original and reduced versions of the Berg Balance Scale and the Western Ontario and McMaster Universities Osteoarthritis Index in patients following hip or knee arthroplasty. Physiother Can.

[28] Berg K, Wood-Dauphinee S, Williams JI (1995). The Balance Scale: reliability assessment with elderly residents and patients with an acute stroke. Scand J Rehabil Med.

[29] Csuka M, McCarty DJ (1985). Simple method for measurement of lower extremity muscle strength. Am J Med.

[30] Peeler J, Anderson JE (2008). Reliability of the Ely's test for assessing rectus femoris muscle flexibility and joint range of motion. J Orthop Res.

[31] Reese NB, Bandy WD (2003). Use of an inclinometer to measure flexibility of the iliotibial band using the Ober test and the modified Ober test: differences in magnitude and reliability of measurements. J Orthop Sports Phys Ther.

[32] Sharma L, Song J, Felson DT, Cahue S, Shamiyeh E, Dunlop DD (2001). The role of knee alignment in disease progression and functional decline in knee osteoarthritis. JAMA.

[33] Malanga GA, Andrus S, Nadler SF, McLean J (2003). Physical examination of the knee: a review of the original test description and scientific validity of common orthopedic tests. Arch Phys Med Rehabil.

[34] Nijs J, Van Geel C, Van der Auwera C, Van de Velde B (2006). Diagnostic value of five clinical tests in patellofemoral pain syndrome. Man Ther.

[35] Fredericson M, Yoon K (2006). Physical examination and patellofemoral pain syndrome. Am J Phys Med Rehabil.

[36] Middleton A, Fritz SL, Lusardi M (2015). Walking speed: the functional vital sign. J Aging Phys Act.

[37] Tevald MA, Murray A, Luc BA, Lai K, Sohn D, Pietrosimone B. Hip abductor strength in people with knee osteoarthritis: A cross-sectional study of reliability and association with function. Knee. 2015. 10.1016/j.knee.2015.06.006 26142154

[38] Costa RA, Oliveira LM, Watanabe SH, Jones A, Natour J (2010). Isokinetic assessment of the hip muscles in patients with osteoarthritis of the knee. Clinics (Sao Paulo).

[39] Hinman RS, Hunt MA, Creaby MW, Wrigley TV, McManus FJ, Bennell KL (2010). Hip muscle weakness in individuals with medial knee osteoarthritis. Arthritis Care Res (Hoboken).

[40] Zeni J, Abujaber S, Pozzi F, Raisis L (2014). Relationship between strength, pain, and different measures of functional ability in patients with end-stage hip osteoarthritis. Arthritis Care Res (Hoboken).

[41] Cooperman JM, Riddle DL, Rothstein JM (1990). Reliability and validity of judgments of the integrity of the anterior cruciate ligament of the knee using the Lachman's test. Phys Ther.

[42] McAlindon TE, Cooper C, Kirwan JR, Dieppe PA (1993). Determinants of disability in osteoarthritis of the knee. Ann Rheum Dis.

[43] Cubukcu D, Sarsan A, Alkan H (2012). Relationships between Pain. Function and Radiographic Findings in Osteoarthritis of the Knee: A Cross-Sectional Study. Arthritis.

[44] Creamer P, Lethbridge-Cejku M, Hochberg MC (2000). Factors associated with functional impairment in symptomatic knee osteoarthritis. Rheumatology (Oxford).

[45] Bohannon RW (2005). Manual muscle testing: does it meet the standards of an adequate screening test?. Clin Rehabil.

[46] Riddle DL, Stratford PW (2013). Unilateral vs bilateral symptomatic knee osteoarthritis: associations between pain intensity and function. Rheumatology (Oxford).

[47] Melchione WE, Sullivan MS (1993). Reliability of measurements obtained by use of an instrument designed to indirectly measure iliotibial band length. J Orthop Sports Phys Ther.

[48] Gulick D (2013). Ortho notes : clinical examination pocket guide.

[49] McClure PW, Rothstein JM, Riddle DL (1989). Intertester reliability of clinical judgments of medial knee ligament integrity. Phys Ther.

[50] Evans PJ, Bell GD, Frank C (1993). Prospective evaluation of the McMurray test. Am J Sports Med.

